# Double trouble, managing bilateral inflammatory breast cancer: a case report

**DOI:** 10.3389/fonc.2025.1595513

**Published:** 2025-08-20

**Authors:** Josep Sabaté-Ortega, Anna Ribera-Montserrat, Sonia del Barco, Ariadna Roqué-Lloveras, Roser Fort-Culillas, M. Carme Carmona-García, Raquel Liñan, Emma Polonio-Alcalá, Gerard Carbó-Vilavedra, Irma Ramos-Oliver, Elisabet Bujons-Buscarons, Claudia Montañés-Ferrer, Gemma Viñas, Helena Pla-Juher

**Affiliations:** ^1^ Department of Medical Oncology, Catalan Institute of Oncology, Dr. Josep Trueta University Hospital, Girona, Spain; ^2^ Precision Oncology Group (OncoGIR-Pro), Girona BiomedicaI Research Institute (IDIBGI-CERCA), Salt, Spain; ^3^ Department of Medical Sciences, Medical School, University of Girona, Girona, Spain; ^4^ Descriptive Epidemiology, Genetic and Cancer Prevention Group, Girona Biomedical Research Institute (IDIBGI-CERCA), Salt, Spain; ^5^ Breast Pathology Unit, IDI Girona Center - Institute of Diagnostic Imaging (IDI), Dr. Josep Trueta University Hospital, Girona, Spain; ^6^ Department of Pathology, Dr. Josep Trueta University Hospital, Girona, Spain

**Keywords:** inflammatory breast neoplasms, bilateral breast neoplasms, multimodal imaging, molecular profiling, therapeutics

## Abstract

Inflammatory breast cancer (IBC) is a rare and aggressive breast cancer type, accounting for 5-7% of breast cancer-related deaths, and its bilateral involvement is exceedingly uncommon. We report a case of metachronous bilateral IBC in a 50-year-old premenopausal woman with Charcot-Marie-Tooth disease, offering novel insight into the diagnostic, therapeutic, and molecular challenges of this condition. The patient initially presented with acute right breast erythema, skin thickening, and *peau d’orange*, followed by contralateral breast involvement with similar symptoms. Disease progression occurred with changes in receptor status and eventual loss of hormone receptor (HR) expression. The initial diagnosis was stage IIIB HR-positive/HER2-negative IBC. The patient underwent neoadjuvant chemotherapy, surgery, adjuvant radiotherapy, and endocrine therapy. However, the patient experienced a contralateral recurrence after 11 months of disease-free survival. Subsequent management involved multiple systemic therapies, including targeted therapy after next-generation sequencing analysis revealed a *PIK3CA* mutation. Although some clinical benefit was achieved, the disease continued to progress. Ultimately, the patient passed away four years after the initial diagnosis. This case underscores the aggressive and recurrent nature of bilateral IBC, its diagnostic complexity, and the importance of molecular profiling in guiding targeted treatment. It highlights the need for clinical vigilance, timely reassessment of tumor biology, and individualized multimodal care in managing rare and evolving presentations of IBC.

## Introduction

Inflammatory breast cancer (IBC) is an invasive rare form of breast cancer (BC) defined by the occurrence of tumor embolism within the dermal lymphatic system (1). Despite its rarity, IBC is responsible for 5.5-7% of BC-related deaths (1, 2). Patients with IBC have a shorter median overall survival (OS) duration compared to those with non-inflammatory BC, with 4.75 years versus 13.40 years for stage III disease (3) and 2.27 years versus 3.40 years for stage IV disease (4).

The diagnosis rates of IBC have been increasing, from 2.0 to 2.5 cases per 100,000 woman-years, leading to a poorer prognosis. In contrast, the rate of non-IBC has declined during the same timeframe. The clinical presentation of IBC can sometimes resemble mastitis, potentially leading to diagnostic delays (5). A distinctive feature in the histopathology of IBC is the infiltration of the dermal lymphatic system, which is responsible for the typical edema and skin alteration that define IBC (5, 6).

There is limited understanding of IBC as a subtype of BC. Ethnicity, race, location, socioeconomic status, obesity, menopausal status, age of menarche, age at first pregnancy, duration of lactation, family history, mammographic density, environmental factors, and genetic factors are risk factors for BC development (7). Notably, higher body mass index (BMI) is associated with increased IBC risk (8), impacting both estrogen receptor (ER)-positive and ER-negative IBC cases in pre- and peri/postmenopausal women (9).

The treatment challenge for IBC arises from its aggressive nature and advanced stage at diagnosis. Unlike other BCs, IBC often lacks a distinct tumor mass, making it hard to target with surgery or radiation therapy. Achieving local control is difficult due to diffuse nature of the disease, and recurrence risk remains high. The treatment typically involves a complex multimodal approach, including chemotherapy (CT), surgery, radiation therapy, and targeted therapies (10).

Bilateral BC refers to the presence of tumors in both breasts and includes two clinical forms. Synchronous cases, observed in 2.9% of cases, involve tumors that appear simultaneously or within 6 months of each other. Metachronous cases, occurring in 4.6% of patients, are characterized by the development of a tumor in the contralateral breast more than 6 months following the primary tumor (11, 12). Despite this, the overall diagnosis of bilateral BC are extremely rare, accounting for only 1% of all breast malignancies (13) and with limited documented cases, making it a unique and challenging condition to treat. Given the aggressive nature of bilateral IBC, early detection and prompt intervention are essential.

Here, we report a unique case of 50-year-old women with Charcot-Marie-Tooth disease diagnosed with IBC hormone receptor(HR)–positive/HER2-negative with metachronous presentation, and secondary loss HR expression after multiple lines of systemic therapy, following the CARE guidelines (**Supplementary Table S1**).

## Case presentation

**Figure 1:**
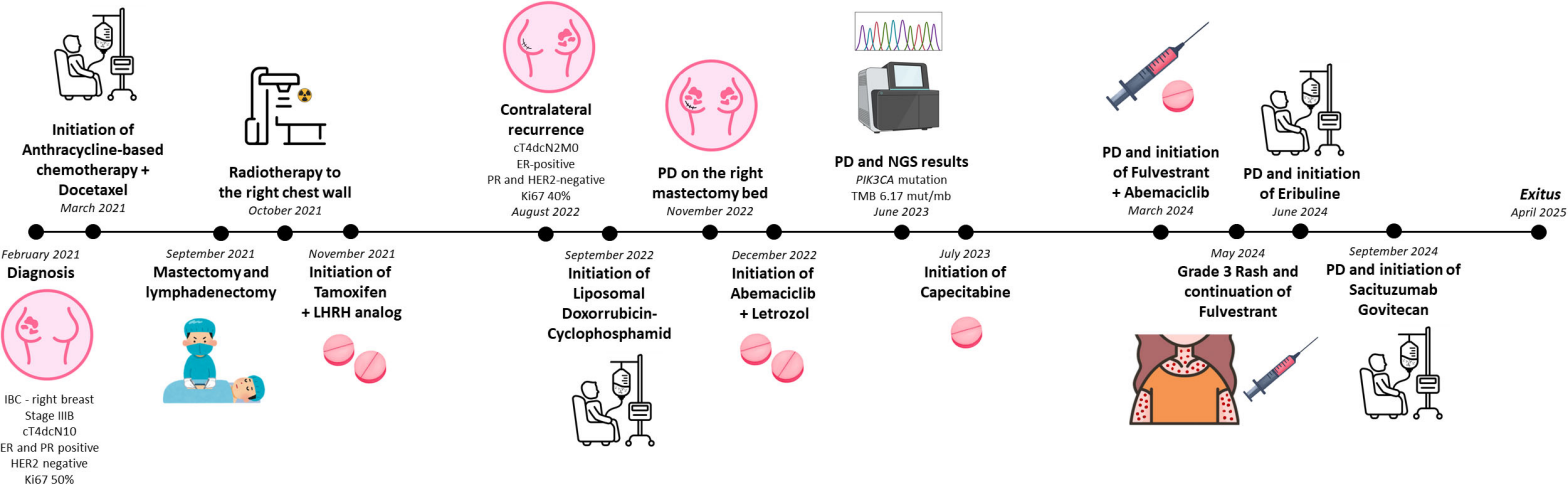
Timeline illustrating the chronological progression of the case. ER, estrogen receptor; HER2, human epidermal growth factor receptor 2; IBC, Inflammatory breast cancer; LHRH, luteinizing hormone-releasing hormone; NGS, next-generation sequencing; PD, progression disease; PR, progesterone receptor.

The progression of the case is summarized in the timeline shown in **Figure 1**.

A 50-year-old premenopausal woman with a known diagnosis with Charcot-Marie-Tooth disease referred to the hospital with acute right breast erythema, skin thickening, and *peau d’orange*. Family history revealed that her mother was diagnosed with BC at the age 45. The patient was referred for genetic counseling; however, no pathogenic germline mutations were identified.

**Figure 2:**
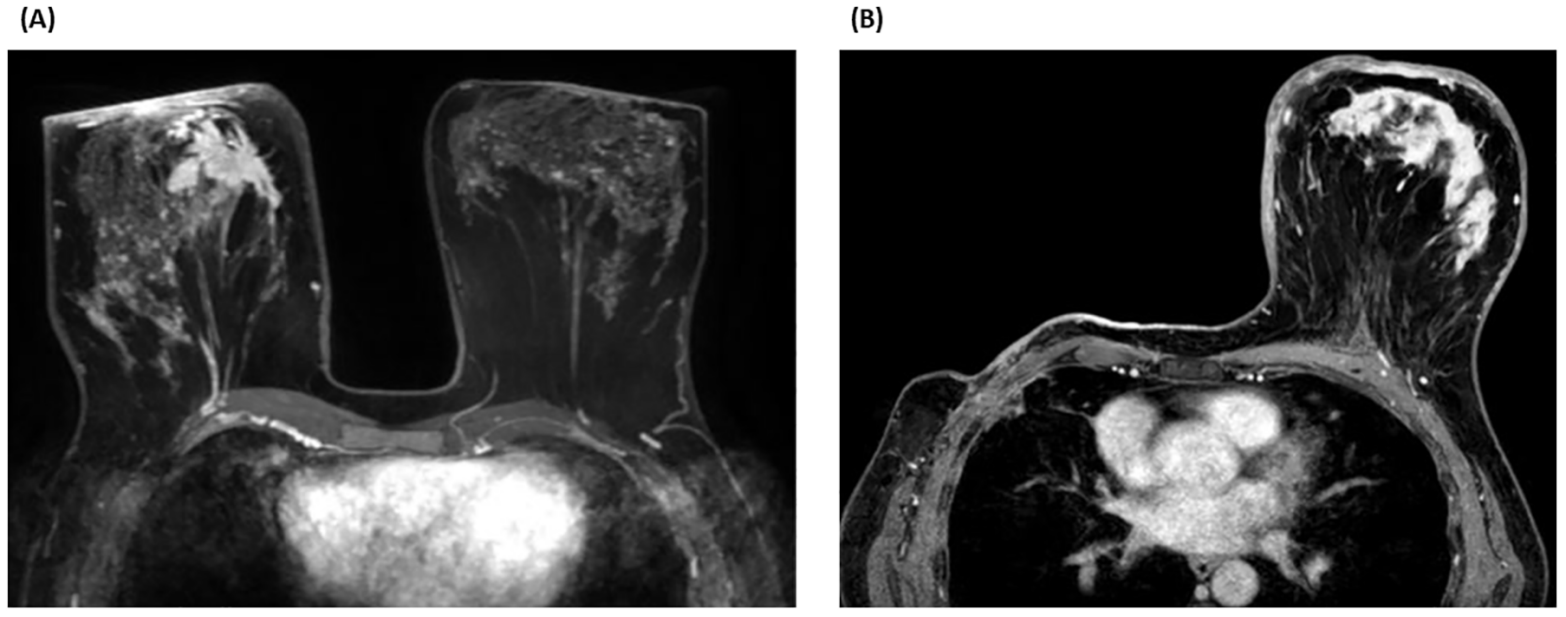
**(A)** (2021) Magnetic resonance imaging (MRI) axial section - T1 sequence with fat suppression and with contrast (phase 2 - 120s). The tumor lesion (41 × 40 × 56 mm) is mainly visualized in the anterior internal plane of the right breast. The chest is flattened in its anterior portion due to the position of the patient. **(B)** (2022) MRI axial section - 3D fat-suppressed T1 sequence (DIXON) with contrast. The skin thickening in the left breast is seen marked with areas of patchy enhancement that could indicate lymphatic metastatic spread and area of enhancement in the central portion of the breast.

Diagnostic imagining included a right breast mammogram, which showed skin thickening and right axillary adenopathy. Magnetic resonance imaging (MRI) revealed a 41 x 40 x 56 mm lesion in the inner quadrant of the right breast, along with diffuse skin thickening and hypercaptating axillary lymph nodes (**Figure 2A**). A body computed tomography (CT) scan showed no evidence of distant metastatic lesions. Core biopsies of both breast lesion and axillary lymph node were performed. Histopathological examination confirmed invasive ductal carcinoma, grade 2, with positive expression of ER and progesterone (PR), HER2 negativity (score 0), and a Ki67 index of 50%. No diagnostic challenges were found. In February 2021, the initial diagnosis was IBC of the right breast, staged as cT4d cN1M0 – stage IIIB, according American Joint Committee on Cancer 8th edition.

In March 2021, the patient initated neoadjuvant CT including four cycles of an anthracycline-based regimen, followed by four cycles of docetaxel administered at 80mg/m^2^ every three weeks. Despite the risk of increased neurotoxicity due to her underlying Charcot-Marie-Tooth disease, systemic treatment was not modified. Instead, the patient was managed in close collaboration with the neurology team to monitor for neurologic side effects throughout the course of treatment.

Surgical intervention was subsequently performed in September 2021. The patient underwent right-sided mastectomy and lymphadenectomy, without breast reconstruction. Histopathological analysis of the surgical specimen revealed a residual invasive ductal carcinoma measuring 9 mm. Of the sixteen lymph nodes resected, one showed micrometastatic tumor deposits (ypT1by pN1mi). Afterwards, the patient received adjuvant radiotherapy directed to the right chest wall, axilla, and internal mammary chain, delivering a total dose of 50 Gy in 25 fractions. Endocrine therapy with tamoxifen (20 mg/day) and luteinizing hormone-releasing hormone (LHRH) analog was initiated thereafter.

**Figure 3:**
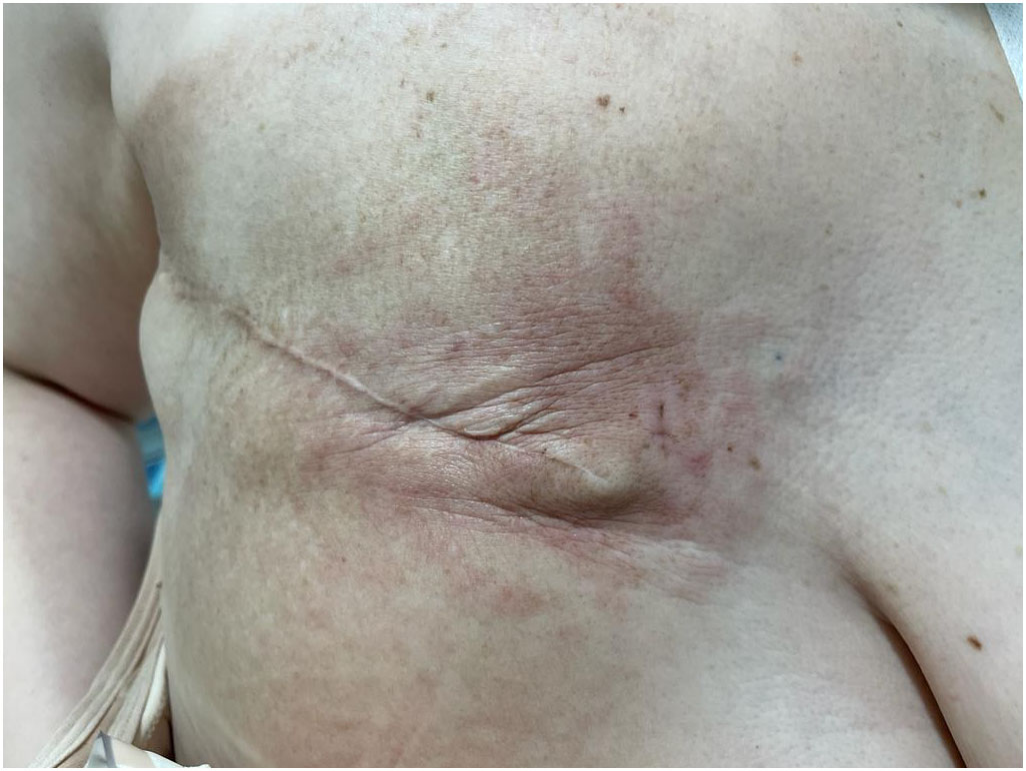
Cutaneous erythema observed over the right mastectomy site.

**Figure 4:**
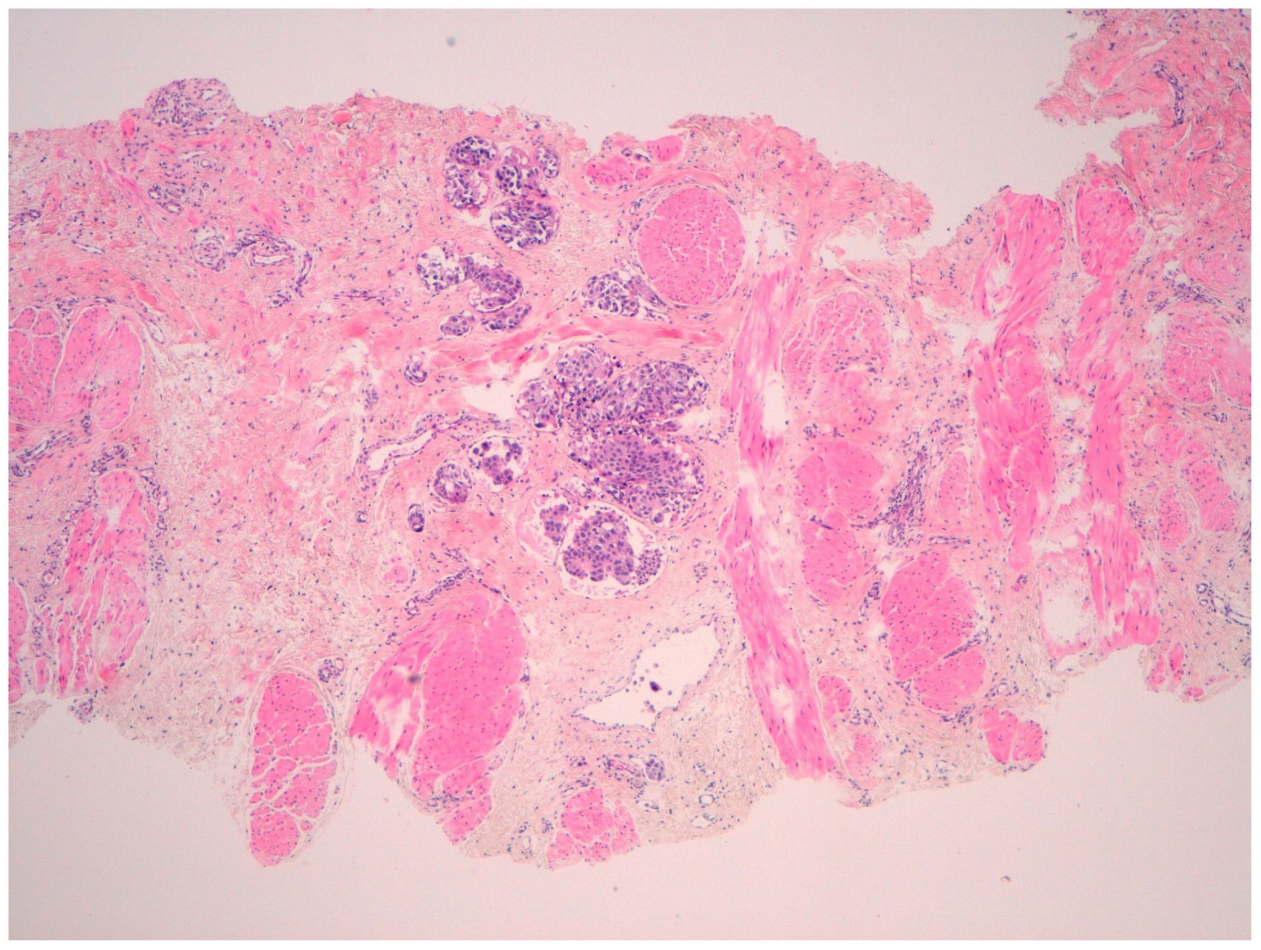
Histopathological analysis of a skin punch biopsy of mammary tissue showing tumor nests within lymphatic vessels at the deep dermal level (hematoxylin stain, 40× magnification).

In August 2022, the patient exhibited erythema in the contralateral (left) breast with palpable fixed axillary lymph nodes, after eleven months of disease-free survival (**Figure 2B**). Biopsies of both the skin and axillary adenopathy revealed invasive ductal carcinoma with ER-positive, PR-negative, HER2-negative (score 0), and Ki67 index of 40%. The recurrence was staged as cT4d cN2 M0 IBC. The patient was treated with a combination of liposomal doxorubicin (60mg/m^2^) and cyclophosphamide (600mg/m^2^) for four cycles. However, the disease progressed, with a new erythema emerging on the right mastectomy bed (**Figure 3**). A repeat biopsy confirmed dermic infiltration by invasive ductal carcinoma with the same molecular profile as the contralateral lesion (**Figure 4**).

First-line treatment for metastatic disease was initiated in December 2022 with a combination of abemaciclib (150 mg/12h) and letrozole (2.5 mg/day). After six months, the disease progressed cutaneously. The next-generation sequencing (NGS) analysis of the tumor identified a *PIK3CA* mutation and a tumor mutational burden (TMB) of 6.17 mutations per megabase. Immunohistochemistry of mismatch repair proteins revealed a stable tumor. As no clinical trial was available for *PIK3CA*-mutant tumors at that time, the patient was started on capecitabine administrated at 1250mg/m^2^ as second-line therapy in July 2023. This regimen resulted in significant clinical improvement in cutaneous symptoms and excellent tolerability for eight months, without significant toxicities.

Upon further locoregional progression, the patient was enrolled in a clinical trial involving fulvestrant (500 mg/month) in combination with alpelisib (300 mg/day) in March 2024. After two cycles, a grade 3 rash was developed, requiring discontinuation of alpelisib. Fulvestrant was continued as a monotherapy but was ineffective; locoregional progression occurred within three months. Eribuline was subsequently introduced as fourth-line treatment, administrated at 1.23 mg/m^2^ on days 1 and 8 of a 21-day cycle. Despite therapy, the disease advanced with development of pleural effusion.

A repeat skin biopsy showed a change in tumor biology: HR-negative invasive carcinoma, HER2 1+, and Ki67 index of 27%. Based on this new profile, in September 2024, fifth-line treatment with sacituzumab govitecan was approved, administrated at 10 mg/kg on days 1 and 8 of a 21-day cycle. The patient received three cycles of treatment; however, it was discontinued due to the onset of respiratory insufficiency. She passed away in April 2025.

## Discussion

This case presentation adds valuable information to the limited literature on bilateral IBC. It offers educational insights about diagnostic challenges and management considerations and raises awareness among clinicians to consider bilateral involvement in patients presenting with breast erythema and edema. By documenting the clinical course, the case also underscores the need for heightened clinical vigilance and may stimulate further research into the pathophysiology and optimal management of this aggressive disease. Although limited by its single-case design and inability to generalize findings or establish causality, it provides meaningful clinical insights into a rare and complex condition.

Bilateral IBC is extremely rare and presents significant challenges to standard treatment due to its aggressive clinical behavior, as illustrated by the case presented. It is worth noting that IBC is usually misdiagnosed as benign conditions like mastitis, which leads to delays in receiving appropriate treatment. With bilateral symptoms, the diagnostic difficulty is particularly pronounced, where a biopsy and imaging workup would be urgent in the setting of persistent symptoms despite antibiotic therapy (5). Owing to its rarity, IBC is underrepresented in clinical trials, resulting in a lack of conclusive evidence regarding optimal treatment and prognosis (14). Reports of synchronous or metachronous contralateral involvement have been documented nearly exclusively in single case reports, underscoring both the extreme rarity of this condition as well as the absence of comprehensive epidemiologic information. In 2009, Masannat et al. described an unusual case of bilateral metachronous IBC, each occurrence showing a complete clinico-pathological response to neoadjuvant CT followed by surgery (15). In 2016, Kurtz et al. reported a patient who received neoadjuvant CT, underwent modified radical mastectomy, and received whole breast radiation for IBC treatment. Less than a year later, the patient experienced a recurrence of IBC on the left chest wall (within the radiated area) and developed a new IBC in the contralateral breast (16). In 2022, Kawaguchi et al. presented a case of contralateral recurrence of triple-negative IBC after 2 years of disease-free survival, successfully managed with dose-dense neoadjuvant CT and bilateral total mastectomy (17). More recently, Levy & Hanna reported a case of HER2-positive IBC with metastatic relapse involving both breasts, the right ovary, and multiple vertebral bodies. The patient achieved a complete response after the treatment with THP (docetaxel, trastuzumab, and pertuzumab) (6).

While these cases emphasize the aggressive nature and recurrence potential of IBC, they also illustrate the clinical variability in disease progression. Of significant note, patients with IBC have a much greater likelihood of developing contralateral breast cancer within two years of diagnosis compared to patients with non-IBC breast cancer (16, 18). Contralateral malignancies detected within two years are more likely to be recurrences or metastases, whereas those diagnosed after 2 years are more often considered independent second tumors (18). In the presented case, contralateral IBC was diagnosed 18 months after the initial diagnosis, fulfilling the criteria for metachronous bilateral BC. This timing also supports the likelihood of metastatic dissemination rather than an independent second tumor. The high metastatic potential of IBC is attributable to several factors, including the intrinsic tumor aggressiveness, specific biological characteristics, the molecular subtype, and demographic risk factors such as African American ethnicity, obesity (especially among postmenopausal women), and advanced age (5, 19, 20).

Imaging techniques such as MRI, CT, positron emission tomography (PET)-CT, mammography, and medical photography are crucial for accurate diagnosis, assessing disease extent and guiding treatment decisions. MRI is the most effective imaging technique for identifying periareolar lesions associated with IBC (5). Both contrast-enhanced and non-contrast MRI can reveal edematous infiltrations, skin thickening, areolar retractions, lymph node enlargement, internal mammary lymphadenopathy, and other features commonly associated with IBC. Dynamic contrast-enhanced MRI, particularly non-contrast T2 weighted imaging (T2WI), is the most sensitive imaging modality for detecting BC and differentiating malignant from benign lesions (21). Bilateral mammography and ultrasound are the initial imaging tools to detect skin thickening, trabecular distortion, increased breast density, or calcifications (19). In addition, PET or CT scans provide valuable information for staging the disease, helping to identify adenopathy, effusion, distant metastases, and locoregional involvement, emphasizing the importance of timely follow-up (22).

The underrepresentation of bilateral IBC in clinical trials poses significant challenges for its management. Treatment is usually based on unilateral IBC and involves a trimodal approach consisting of neoadjuvant CT followed by modified radical mastectomy and radiotherapy (19, 23). However, bilateral involvement adds additional complexity to surgical planning and the design of radiation treatment fields. Active monitoring using dynamic imaging techniques along with hands-on evaluations post therapeutic interventions is essential due to IBC’s aggressive nature and elevate recurrence risk to timely identify disease advancement—enhancing survival chances (10). Treatment guidance using comprehensive molecular profiling becomes invaluable for identifying clinically relevant alterations like *HER2* amplification or *PIK3CA* mutations enabling integration of targeted therapies or endocrine treatments (24, 25). Efforts to advance immune checkpoint inhibitor-based treatments have also prompted numerous studies investigating the expression of various immune checkpoints in IBC. Given the success of PD-1/PD-L1 inhibitors in other cancers, current research has predominantly focused on PD-L1 expression in IBC (24, 26).

Genomic profiling is essential for understanding the complex molecular landscape of IBC, allowing the identification of specific genetic alterations, such as copy number variations or mutations (27). It also enables the identification of predictive factors for treatment response by analyzing gene expression signatures associated with CT response, thereby facilitating personalized treatment strategies (27). Moreover, genomic profiling helps to elucidate candidate genes and pathways implicated in IBC progression, including cell proliferation, invasion, and immune response, contributing to a better understanding of the underlying molecular features of IBC, with the aim of improving the diagnosis and treatment of this aggressive form of BC. For instance, Chakraborty et al. identified that EMT scores showed a higher coefficient of variation in IBC compared to non-IBC samples, suggesting it as a potential biomarker (25).

In the case presented, we identified a mutation in *PIK3CA* through NGS analysis, which allowed the patient to receive fulvestrant in combination with alpelisib. The PI3Kα inhibitor alpelisib has demonstrated significant clinical benefit when combined with endocrine therapy in patients with HR-positive/HER2-negative advanced breast cancer harboring *PIK3CA* mutations (28). Approximately 40% of HR+/HER2− BC metastases harbor mutations in the PI3K–AKT–mTOR pathway, which diminish endocrine therapy efficacy and promote resistance. Among these, *PIK3CA* mutations are the most prevalent, markedly boosting this pro-survival signaling cascade (29). In the pivotal SOLAR-1 trial, alpelisib plus fulvestrant almost doubled median progression-free survival compared to fulvestrant alone in patients with *PIK3CA*-mutated tumors (11.0 vs. 5.7 months; HR 0.65; P < 0.001) (28). This improvement was both statistically and clinically significant, establishing the PI3K pathway as a viable target in this context. As a result, alpelisib received FDA approval in 2019 for use with endocrine therapy in postmenopausal women and older men with HR+/HER2− advanced BC with *PIK3CA* mutations following progression on prior endocrine therapy (30). This combination now represents a genomically guided standard of care that enhances disease control by addressing a key mechanism of resistance. In this context, confirming a *PIK3CA* mutation in tumor tissue supports the use of the PI3K inhibitor alpelisib to improve disease control and overcome endocrine resistance, exemplifying the principles of precision oncology.

From the patient’s perspective, she conveyed significant uncertainty about her future following treatment for metastatic BC. She reported struggling with body image and distress stemming from the side effects of therapy, which adversely affected her self-esteem and daily functioning. In recognition of the emotional and psychological burden of her illness, she was referred to the psycho-oncology services for specialized support. Notably, she was sustained by a strong familial and social support network, which offered significant emotional support and resilience throughout her illness.

## Conclusions

Our case report underscores the aggressive and diagnostically challenging nature of IBC, particularly in its rare bilateral, metachronous presentation. It emphasizes the necessity for heightened clinical vigilance, especially when symptoms mimic benign conditions, and the critical role of timely biopsy and imaging. The case demonstrates how tumor biology can evolve under therapeutic pressure, requiring repeated reassessment through regular and consistent imaging and physical examinations to inform treatment decisions. It also emphasize the importance of patient adherence to prescribed therapy, as consistent compliance significantly influences treatment efficacy and outcomes. Furthermore, it exemplifies the value of molecular profiling in informing precision oncology, as demonstrated by the identification of a *PIK3CA* mutation. Early and accurate detection, along with personalized, multimodal therapeutic approach, can significantly improve the chances of successful therapy and long-term survival. Ultimately, this case contributes meaningful insight into a rare and complex oncological entity and the ongoing need for comprehensive, individualized care.

## Data Availability

The original contributions presented in the study are included in the article/**Supplementary Material**. Further inquiries can be directed to the corresponding authors.
